# Anthranilate Acts as a Signal to Modulate Biofilm Formation, Virulence, and Antibiotic Tolerance of Pseudomonas aeruginosa and Surrounding Bacteria

**DOI:** 10.1128/spectrum.01463-21

**Published:** 2022-01-12

**Authors:** Hyeon-Ji Hwang, Xi-Hui Li, Soo-Kyoung Kim, Joon-Hee Lee

**Affiliations:** a Department of Pharmacy, College of Pharmacy, Pusan National University, Busan, South Korea; University of Guelph

**Keywords:** *Pseudomonas aeruginosa*, anthranilate, anthranilate peak, biofilm, antibiotic tolerance, virulence, signaling molecule

## Abstract

Anthranilate is a diffusible molecule produced by Pseudomonas aeruginosa and accumulates as P. aeruginosa grows. Anthranilate is an important intermediate for the synthesis of tryptophan and the Pseudomonas quinolone signal (PQS), as well as metabolized by the anthranilate dioxygenase complex (*antABC* operon products). Here we demonstrate that anthranilate is a key factor that modulates the pathogenicity-related phenotypes of P. aeruginosa and other surrounding bacteria in the environment, such as biofilm formation, antibiotic tolerance, and virulence. We found that the anthranilate levels in P. aeruginosa cultures rapidly increased in the stationary phase and then decreased again, forming an anthranilate peak. Biofilm formation, antibiotic susceptibility, and virulence of P. aeruginosa were significantly altered before and after this anthranilate peak. In addition, these phenotypes were all modified by the mutation of *antABC* and exogenous addition of anthranilate. Anthranilate also increased the antibiotic susceptibility of other species of bacteria, such as Escherichia coli, Salmonella enterica, Bacillus subtilis, and Staphylococcus aureus. Before the anthranilate peak, the low intracellular anthranilate level was maintained through degradation from the *antABC* function, in which induction of *antABC* was also limited to a small extent. The premature degradation of anthranilate, due to its high levels, and *antABC* expression early in the growth phase, appears to be toxic to the cells. From these results, we propose that by generating an anthranilate peak as a signal, P. aeruginosa may induce some sort of physiological change in surrounding cells.

**IMPORTANCE** Pseudomonas aeruginosa is a notorious pathogen with high antibiotic resistance, strong virulence, and ability to cause biofilm-mediated chronic infection. We found that these characteristics change profoundly before and after the time when anthranilate is produced as an “anthranilate peak”. This peak acts as a signal that induces physiological changes in surrounding cells, decreasing their antibiotic tolerance and biofilm formation. This study is important in that it provides a new insight into how microbial signaling substances can induce changes in the pathogenicity-related phenotypes of cells in the environment. In addition, this study shows that anthranilate can be used as an adjuvant to antibiotics.

## INTRODUCTION

Pseudomonas aeruginosa, an opportunistic human pathogen, is of great interest because of its remarkable metabolic versatility and ability to infect a variety of hosts. P. aeruginosa produces various metabolites, such as phenazines, pyocyanin, quinolones, acyl-homoserine lactones, anthranilate, and so on, and most of them are secreted into the environment (1, 2). One of them, anthranilate, is an important intermediate for the synthesis of tryptophan and the Pseudomonas quinolone signal (PQS), or is otherwise degraded by the anthranilate dioxygenase complex via the TCA cycle (Fig. S1 in the supplemental material) (2–4). Therefore, anthranilate is a key metabolite of P. aeruginosa at the metabolic branch point, and a substance that can affect the surrounding environment and cells (2, 5). Nevertheless, there has not been much research on the effect of anthranilate on the physiology and pathogenicity of P. aeruginosa, whereas the products of anthranilate metabolism, such as PQS and tryptophan, have been studied a lot in regard to biofilm formation and virulence of this pathogen (6, 7).

Recent studies have shown that anthranilate accumulates in high concentrations at stationary phase (2). At the same time, the *antABC* operon encoding the anthranilate dioxygenase complex, which degrades anthranilate, is rapidly induced because anthranilate is an inducer for *antABC* expression (2, 5). AntR, a transcriptional regulator, induces *antABC* by recognizing anthranilate and binding to the *antABC* promoter (4). However, besides this direct regulation, the anthranilate production and expression of *antABC* in P. aeruginosa are complicatedly regulated by quorum sensing (QS), a cell-to-cell communication system, and some peculiar, difficult to understand results have been reported about this QS regulation so far, as described below.

Anthranilate is normally produced in *lasR* mutants, but not produced in *rhlR* mutants (2). In addition, while the expression of *antABC* is regulated by QS signals, acyl-homoserine lactones (acyl-HSLs), this regulation occurs in a QS signal receptor (LuxR homologs)-independent manner (8). This means that the QS signal works without its own receptor, suggesting that there is a mechanism that we do not yet understand. One study on the MexGHI-OpmD efflux pump that is responsible for the production of acyl-HSLs and PQS in P. aeruginosa, also showed another interesting but difficult to understand result. The mutant of this pump had impaired growth and altered antibiotic resistance and virulence, which was presumed to be due to the accumulation of anthranilate or its metabolic derivative(s) (9). However, anthranilate is a substance that is normally secreted and accumulated in the stationary phase when P. aeruginosa grows (2), so it is not clear how this impairs growth.

Another peculiar result about anthranilate came from a biofilm study. P. aeruginosa is a bacterium that forms biofilms very well (10). However, anthranilate was reported to have an antibiofilm effect on various bacteria by causing biofilm dispersal (11–13). Since anthranilate accumulates to a high level in stationary phase (2), it is questionable how P. aeruginosa can form well-made biofilms in the presence of high levels of anthranilate. A recent study showed that P. aeruginosa in biofilms have a high expression of *antABC* (14), implying that these cells have enhanced anthranilate degrading activity. We therefore hypothesized that the anthranilate level should be reduced before P. aeruginosa begins to form a biofilm. In order to answer this question, this study was intended to pursue how the level of anthranilate changes as P. aeruginosa grows and when P. aeruginosa changes its life mode to a biofilm state. In addition, since relatively few studies have been conducted on anthranilate regarding the pathogenicity-related phenotypes of P. aeruginosa, we also investigated how it is involved in antibiotic resistance and virulence. Our results showed that anthranilate rapidly increased in the stationary phase and then decreased in a peak shape, and the biofilm formation occurred after this anthranilate peak. In addition, antibiotic tolerance and virulence of P. aeruginosa increased after the anthranilate peak. Because anthranilate also affected these characteristics in other bacteria (12), we suggest that anthranilate acts as a signaling molecule that modulates P. aeruginosa, neighboring cells, and eventually the microbial community.

## RESULTS

### The “anthranilate peak” is generated by *antABC* in the stationary phase.

**FIG 1:**
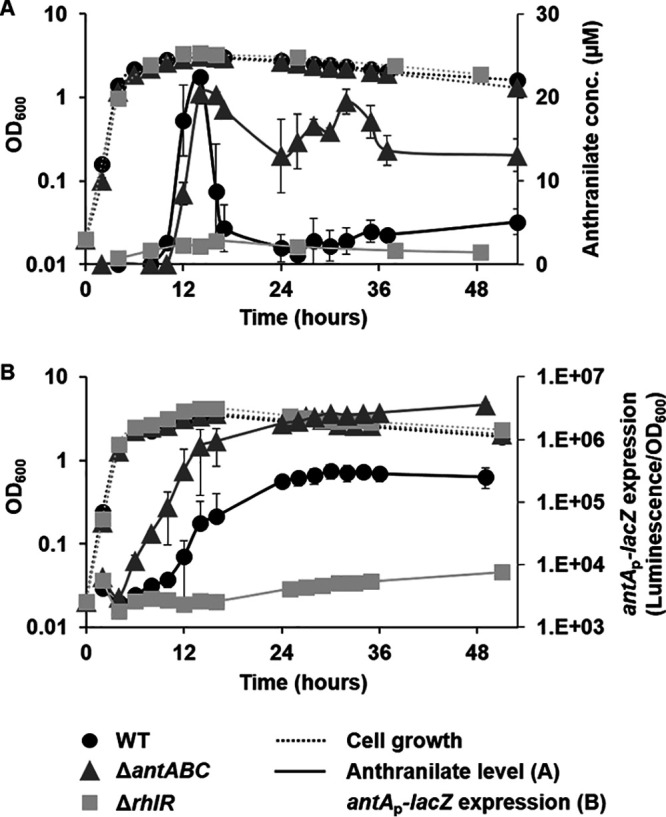
The “anthranilate peak” and *antABC* expression. (A) P. aeruginosa PAO1 (WT), *antABC* mutant (Δ*antABC*), and *rhlR* mutant (Δ*rhlR*) were grown for 53 h in LB broth, and the anthranilate levels in culture media were measured by an E. coli reporter strain. (B) The expression of *antA_p_-lacZ* in P. aeruginosa was measured through growth using the reporter plasmid (pJL201).

To test our hypothesis that the anthranilate level should decrease before P. aeruginosa begins to form a biofilm, we traced the change of the anthranilate level in culture medium as P. aeruginosa grows, by using a reporter strain (Fig. 1A) and HPLC (Fig. S2) analyses. As previously reported (2), the production and secretion of anthranilate remain very low until P. aeruginosa enters the stationary phase, then it begins to secrete and rapidly accumulate to a high level later in the stationary phase. However, the level of anthranilate decreased again as the stationary phase persisted (Fig. 1A, Fig. S2). This transient increase in the anthranilate level appeared as an “anthranilate peak” that reached the highest point at 14 h from the start of cultivation. In order to know whether this decrease of anthranilate is driven by the *antABC* operon, we constructed an *antABC*-deleted mutant (Δ*antABC*, Fig. S3) and measured the anthranilate levels. The result showed that the accumulation of anthranilate remains elevated in the *antABC* mutant (Fig. 1A), indicating that the decrease in the anthranilate level was mostly driven by *antABC* function. In our previous study, we found that *rhlR* mutants do not produce anthranilate (2). The anthranilate levels in our *rhlR* mutant were very low throughout growth and did not have an anthranilate peak (Fig. 1A).

When we examined the expression of *antABC* in the wild type using *antA*_p_-*lacZ* fusion, it was expressed at very low levels in the early exponential phase and appeared to be induced when anthranilate began to accumulate (Fig. 1B). However, if the graph is converted into a logarithmic scale in order to examine precisely when induction begins, it clearly shows that the induction of *antABC* expression begins somewhat earlier than the accumulation of anthranilate (Fig. 1B). This result suggests that even before anthranilate is secreted outside the cell, its concentration in the cytoplasm already increases high enough to activate AntR, the regulator of *antABC*. The *antA*_p_-*lacZ* was induced much earlier in the *antABC* mutant (Fig. 1B), indicating that anthranilate increased rapidly in the mutant cells. This means that anthranilate is maintained at low levels during the early phase of growth, not only because anthranilate is rapidly consumed for PQS or tryptophan synthesis, but also because a significant part of it is removed through degradation. The expression of *antA_p_-lacZ* in both the wild type and *antABC* mutant did not decrease even after the decrease in extracellular anthranilate (Fig. 1B). This suggests that there is still a sufficient concentration of anthranilate within the cell. Therefore, we directly investigated the intracellular anthranilate levels using thin-layer chromatography (TLC) analysis, which showed the continuous increase of anthranilate within the cells (Fig. S4). The *antABC* operon was not induced in the *rhlR* mutant (Fig. 1B), confirming that anthranilate is not significantly produced in *rhlR* mutants.

In conclusion, extracellular anthranilate increases rapidly in the stationary phase and then decreases again to form an anthranilate peak, whereas within cells anthranilate is accumulated at a considerable level even after the peak, all while *antABC* is induced according to this increase in anthranilate in order to regulate its level.

### The anthranilate peak hinders biofilm formation of P. aeruginosa.

**FIG 2:**
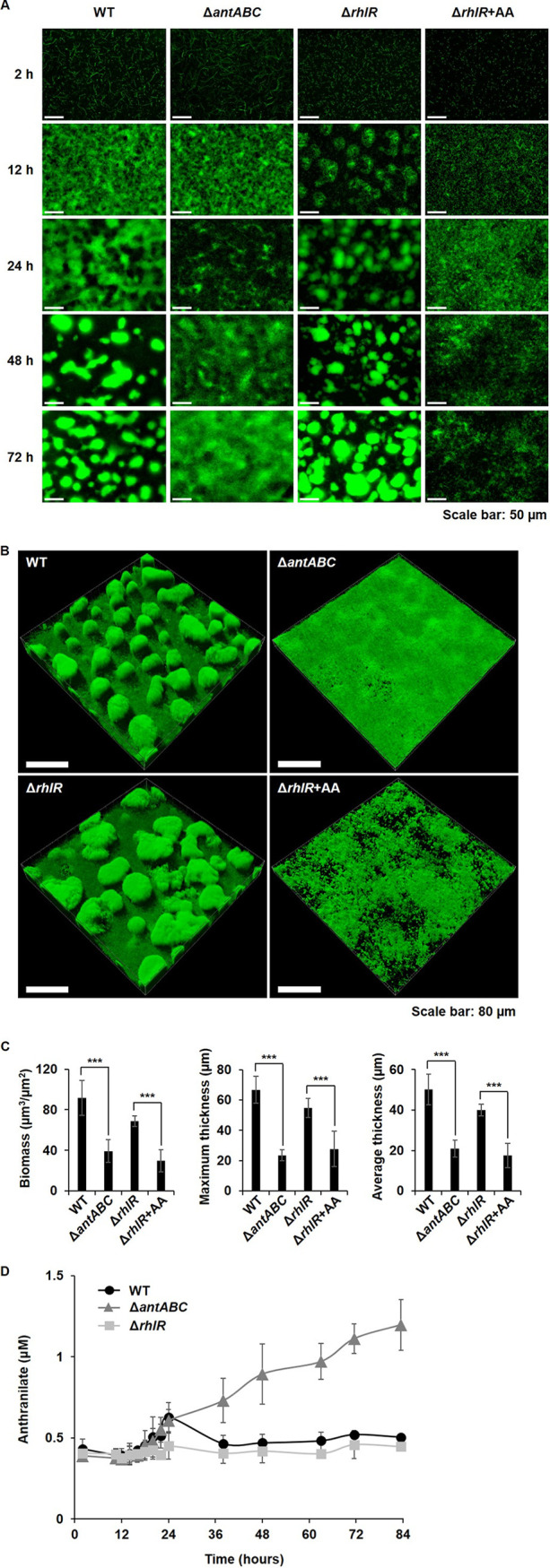
Anthranilate production and biofilm formation of P. aeruginosa. P. aeruginosa PAO1 (WT), *antABC* mutant (Δ*antABC*), and *rhlR* mutant (Δ*rhlR*) harboring the GFP-expressing plasmid (pAB1) were grown in flow-cell chambers for 84 h in order to form biofilms. +AA indicates the addition of 0.1 mM anthranilate. (A) The process of biofilm growth was observed at the indicated time points with a fluorescence microscope. (B) The 3-D images of biofilm structure were obtained by CLSM at 84 h. (C) The biomass and thickness of the biofilms in B were quantified by COMSTAT. ***, *P < *0.005. (D) During biofilm growth, anthranilate levels in the spent medium from the flow-cells were measured by using the reporter strain.

Since anthranilate has an antibiofilm effect, the high-level production of anthranilate in the *antABC* mutant was hypothesized to negatively affect biofilm formation. The static biofilm assay showed that the *antABC* mutant formed less biofilm than the wild type, which was similar to the results of wild-type biofilm treated with anthranilate or another well-known biofilm inhibitor, sodium nitroprusside (Fig. S5). When we investigated the timing of the biofilm formation of P. aeruginosa in a flow-cell system, the wild type began to form a biofilm structure in about 48 h, but the *antABC* mutant did not form a biofilm structure even after 84 h (Fig. 2A and B). In the *rhlR* mutant, the initial microcolonies and stalks formed earlier than in the wild type (before 24 h; Fig. 2A). This is a noticeable result in that the *rhlR* mutant barely produces rhamnolipids that are proposed to be required for biofilm development (15–18). The *rhlR* mutant produced a well-formed biofilm, but when anthranilate was added to it, the biofilm collapsed (Fig. 2A). The 3-D structure of the biofilms also showed similar results (Fig. 2B and C).

We assessed anthranilate levels in the flow-cells during biofilm growth by measuring anthranilate in the drains from the flow-cells. As observed in the planktonic culture, the anthranilate peak was observed at about 24 h in the wild type, and was modified in the *antABC* and *rhlR* mutants (Fig. 2D). The high anthranilate level was maintained without a decrease in the *antABC* mutant, and anthranilate did not accumulate in the *rhlR* mutant. Compared to the biofilm result at 48 h (Fig. 2A), this result confirms that the structural differentiation of the biofilm begins after the anthranilate peak. Taken together, it is obvious that the endogenous production of anthranilate has an important influence on biofilm formation.

### The virulence factor production of P. aeruginosa changes before and after the anthranilate peak.

**FIG 3:**
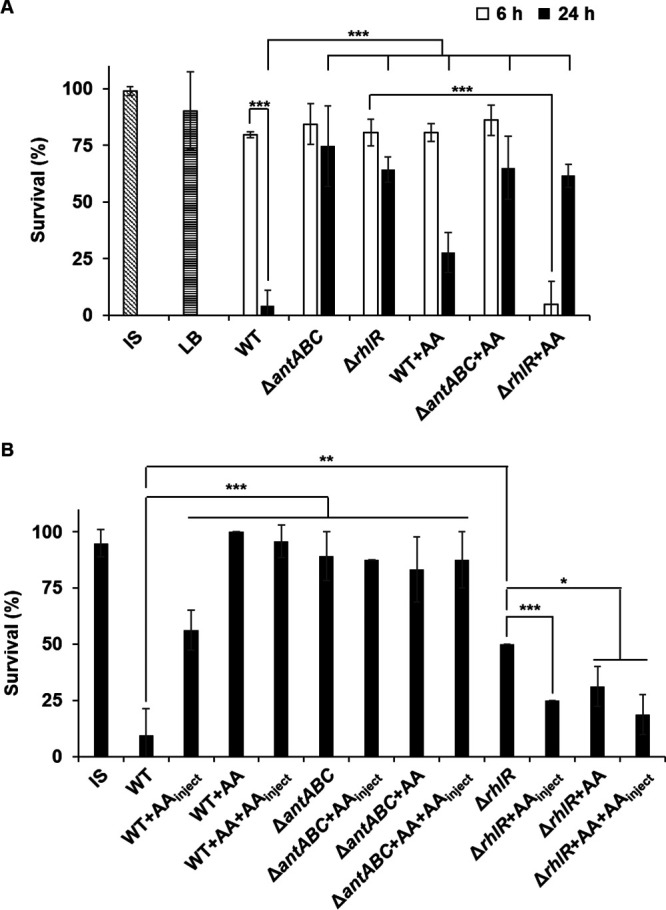
Comparison of virulence factor production before and after the anthranilate peak. (A) The culture supernatants were prepared from each strain at 6 or 24 h, and injected into *T. molitor* larvae. Insect saline (IS) and LB were injected as controls. (B) 5.6 × 10^2^ CFU of P. aeruginosa wild-type cells were resuspended in IS and injected into *T. molitor* larvae. The larvae were incubated at 25°C for 2 days and the live/dead were counted. +AA indicates the addition of 0.1 mM anthranilate to culture media during cultivation and +AA_inject_ indicates the co-injection of 0.1 mM anthranilate into larvae. *, *P < *0.05; **, *P < *0.01; ***, *P < *0.005.

To figure out whether other physiological phenotypes are altered by the anthranilate peak, the virulence factor production of P. aeruginosa was compared before and after the peak (at 6 and 24 h in Fig. 1A, respectively) by measuring the toxicity of culture supernatants to *Tenebrio molitor* larvae (19, 20). Although cells at 6 and 24 h were both in the stationary phase (Fig. 1A), the toxicity was much higher at 24 h than at 6 h (Fig. 3A), indicating that the production of virulence factors increases after the anthranilate peak. The toxicity of the culture supernatant from the *antABC* mutant significantly decreased at both the 24 and 6 h time points (Fig. 3A). This reduced production of virulence factors in the *antABC* mutant is somewhat unexpected because without *antABC*, PQS synthesis would theoretically increase, therefore leading to an increase in virulence. Thus, we thought that the reduced production of virulence factors may be caused by the presence of anthranilate in a high concentration after the peak. To confirm this, anthranilate was added to the medium from the beginning of the culture, and this exogenous anthranilate significantly reduced the virulence factor production of the wild type at 24 h (Fig. 3A). This is probably because anthranilate is not sufficiently degraded and remained at a high concentration even after 24 h like the *antABC* mutant.

We also investigated whether P. aeruginosa was actually attenuated in the presence of anthranilate, by injecting live P. aeruginosa cells into *T. molitor* larvae. In this way, anthranilate in culture media is diluted when diluting bacteria for injection and the injected cells would multiply in the host during pathogenesis, so the physiological state at the specific growth stage might be reset in the host. Therefore, the experiments were carried out in cases where anthranilate was not added at all, anthranilate was added only during culture (+AA), anthranilate was added only at injection (+AA_inject_), and anthranilate was added both during culture and at injection (+AA+AA_inject_). Our results showed that the P. aeruginosa cells grown in the presence of anthranilate showed decreased virulence (Fig. 3B). Interestingly, the cells injected with anthranilate were also significantly attenuated (Fig. 3B). The *antABC* mutant that accumulates anthranilate showed decreased virulence regardless of exogenous anthranilate (Fig. 3B). This result suggests that anthranilate is involved in virulence and the degradation of anthranilate may be important.

Both the virulence factor production and actual virulence of the *rhlR* mutant were also attenuated (Fig. 3A and B), as has already been reported elsewhere (18), yet we discovered an unexpected result regarding anthranilate. Until now, it has been thought that the *rhlR* mutant is attenuated because virulence factors that are regulated by RhlR are not expressed. Thus, the *rhlR* mutant was expected to have low virulence regardless of anthranilate. However, the addition of anthranilate dramatically increased the virulence factor production of the *rhlR* mutant at 6 h, although it didn’t increase at 24 h (Fig. 3A). When the live *rhlR* mutant cells were injected, the virulence also increased with anthranilate (Fig. 3B). This result implies that there is something other than the genes regulated by RhlR causing virulence and anthranilate is required. Rather, early in growth, RhlR seems to inhibit the expression of this anthranilate-mediated virulence, because the wild type does not produce virulence factors with the early addition of anthranilate (Fig. 3A). All of these results show that virulence is altered by both the production and degradation of anthranilate.

### The anthranilate peak modulates the antibiotic tolerance of P. aeruginosa.

**FIG 4:**
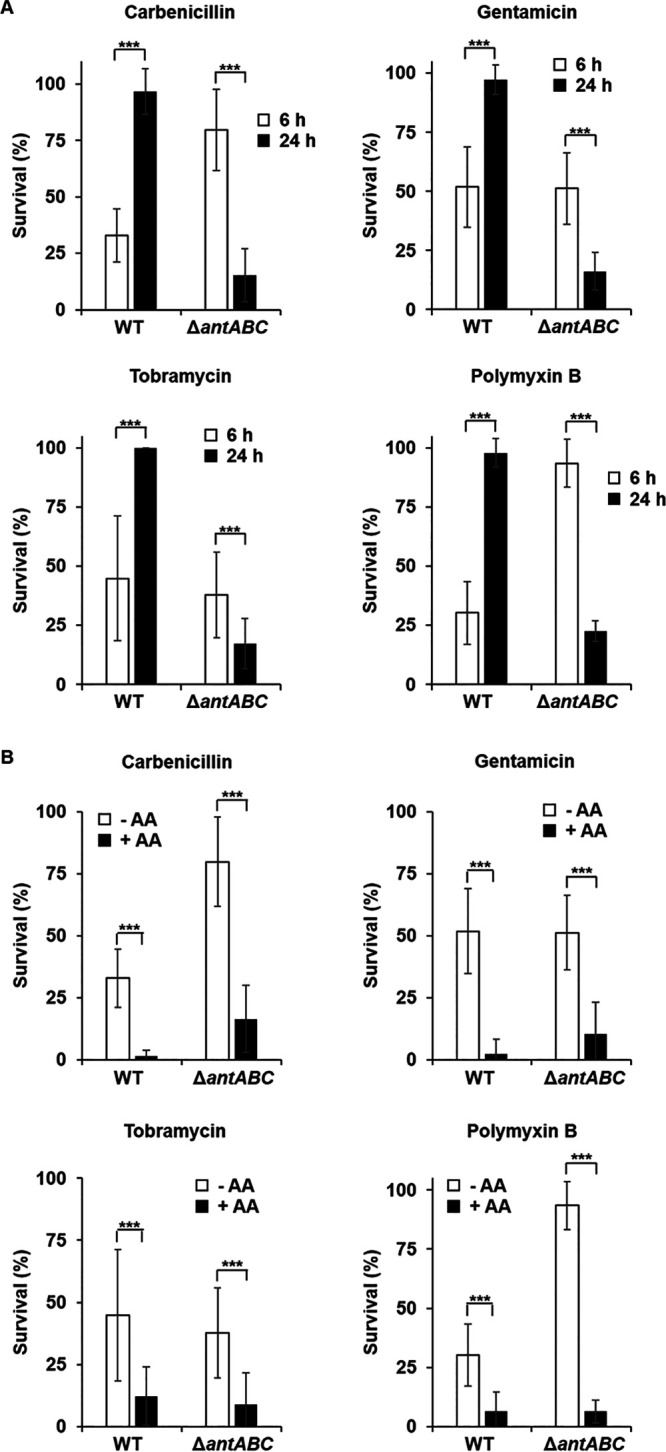
Comparison of antibiotic susceptibility before and after the anthranilate peak. P. aeruginosa PAO1 WT and the *antABC* mutant (Δ*antABC*) were grown in LB medium without anthranilate or with 0.1 mM anthranilate. Cells were taken at 6 or 24 h, diluted in PBS, and exposed to antibiotics for 2 h. The surviving cells were tallied by viable counting and each count was normalized to the sample without antibiotics (which corresponds to 100%). The antibiotic susceptibility was compared between cells taken from the 6- or 24-h culture without anthranilate (A) or between cells taken from the 6-h culture with (+AA) or without anthranilate (–AA) (B) antibiotics were used at the following concentrations; carbenicillin, 50 μg/mL; gentamicin, 1 μg/mL; tobramycin, 1 μg/mL; polymyxin B; 1 μg/mL. ***, *P < *0.005.

Usually, when the stationary phase continues and cells become dormant, susceptibility to antibiotics decreases. Interestingly, however, when we compared the antibiotic susceptibility of P. aeruginosa before and after the anthranilate peak, antibiotic susceptibility of the wild type decreased, while the *antABC* mutant showed an increase in susceptibility to various antibiotics after the anthranilate peak (Fig. 4A). In this experiment, we used four different antibiotics, carbenicillin, gentamicin, tobramycin, and polymyxin B, that are commonly used in the treatment of P. aeruginosa infections (21, 22). Our results signify that if anthranilate is not properly degraded and kept at a high concentration, susceptibility to antibiotics increases. To see if anthranilate really alters the antibiotic susceptibility of P. aeruginosa, we added anthranilate to media exogenously, and this early addition of anthranilate increased the susceptibility to antibiotics in both the wild type and *antABC* mutant (Fig. 4B). These results show that there is anthranilate-mediated change in antibiotic susceptibility that was not previously known.

In this experiment, we measured the degree of survival after antibiotic treatment rather than measuring the change in the MIC. This was because bacterial cells must be cultured again to measure the MIC, which resets the physiological state of the cells at 6 and 24 h. In fact, when we cultured the wild type and *antABC* mutant with or without anthranilate and measured the MIC with cells at 6 and 24 h, there was no significant difference with the exception of the *antABC* mutant having an increased MIC to carbenicillin compared to the wild type (Fig. S6). Recently, resistance and tolerance to antibiotics have been defined differently: resistance is defined as the inheritable ability of a cell to grow in the presence of an antibiotic, whereas tolerance is defined as the ability of a bacterial population to survive a short-term exposure to an antibiotic and does not cause a change in their MIC (23). Therefore, our results show the change in tolerance rather than resistance to antibiotics.

We note that the *antABC* mutant showed decreased susceptibility to carbenicillin and polymyxin B at 6 h, but not to gentamicin and tobramycin (Fig. 4B). Although the exact mechanism is not known, this result also shows that the anthranilate metabolism and antibiotic tolerance are somehow related.

### Anthranilate increases the susceptibility of other bacteria to antibiotics.

**FIG 5:**
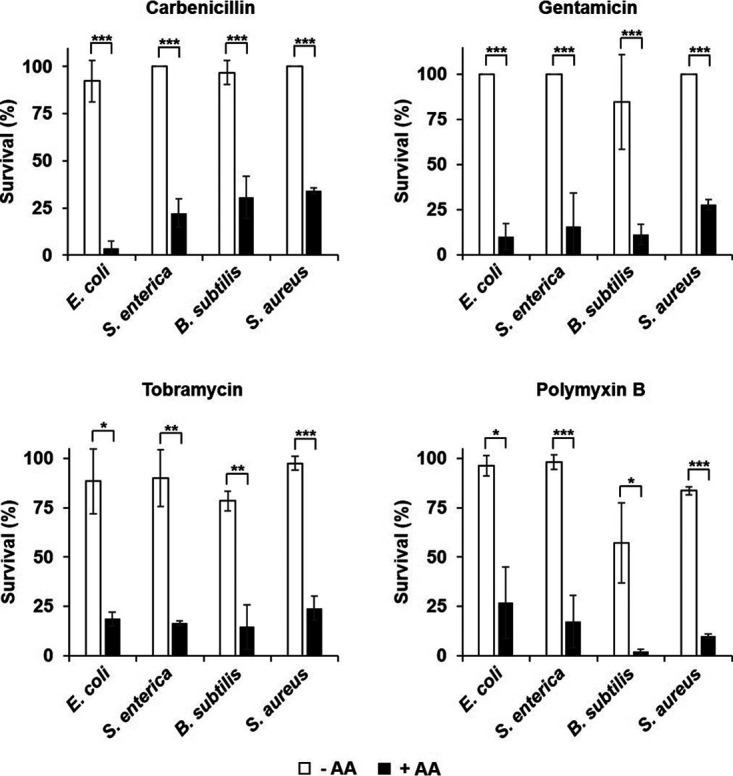
Increase of antibiotic susceptibility by anthranilate. E. coli, S. enterica, B. subtilis, and S. aureus were grown without anthranilate or with 0.1 mM anthranilate. The bacterial cells were taken at OD_600_ = 2.0, diluted in PBS, and exposed to antibiotics for 2 h. The surviving cells were tallied by viable counting and each count was normalized to the sample without antibiotics (which corresponds to 100%). Antibiotics were used at the following concentrations: carbenicillin, 1 μg/mL (for E. coli, S. enterica, and B. subtilis), and 5 μg/mL (for S. aureus); gentamicin, 1 μg/mL (for E. coli and S. aureus), and 5 μg/mL (for S. enterica and B. subtilis); tobramycin, 0.5 μg/mL (for E. coli), 1 μg/mL (for B. subtilis *and* S. aureus), and 5 μg/mL (for S. enterica); polymyxin B, 0.5 μg/mL (for E. coli), 5 μg/mL (for S. enterica), and 50 μg/mL (for B. subtilis *and* S. aureus). *, *P < *0.05; **, *P < *0.01; ***, *P < *0.005.

Previously, we have reported that anthranilate has an antibiofilm effect on other bacteria (12), which means that the anthranilate secreted by P. aeruginosa can affect the physiology of other bacteria around it. Since the anthranilate addition increases the sensitivity of P. aeruginosa to multiple antibiotics, we were curious if anthranilate had a similar effect on antibiotic susceptibility of other bacteria. We tested four different bacteria for this effect by cultivating with or without anthranilate and treating with antibiotics, and the results showed that anthranilate increased the susceptibility to antibiotics in all bacteria tested (Fig. 5). We don’t know the exact mechanism underlying this phenomenon, but this result demonstrates that anthranilate produced by P. aeruginosa can affect other bacteria in a multi-species bacterial community.

### Early degradation of anthranilate is likely toxic to cells.

Fig. 1A showed that *antABC* is likely to be the only gene responsible for anthranilate degradation in P. aeruginosa. We confirmed this from the absence of growth in the *antABC* mutant in MMA, a minimal medium containing anthranilate as the sole carbon source (Fig. S7AB). In addition, we found that even the wild type had a long lag period of growth at the beginning of the culture in MMA (Fig. S7A). More precisely, it looked like an intermediate lag that was growing but then quickly plateaued until approximately 32 h before resuming again. During this lag, the added anthranilate did not initially decrease, but it began to decrease as growth restarted after the lag (Fig. S7A). This result suggests that degradation of anthranilate in the early stage of growth may not be favorable for cells and is strongly repressed.

**FIG 6:**
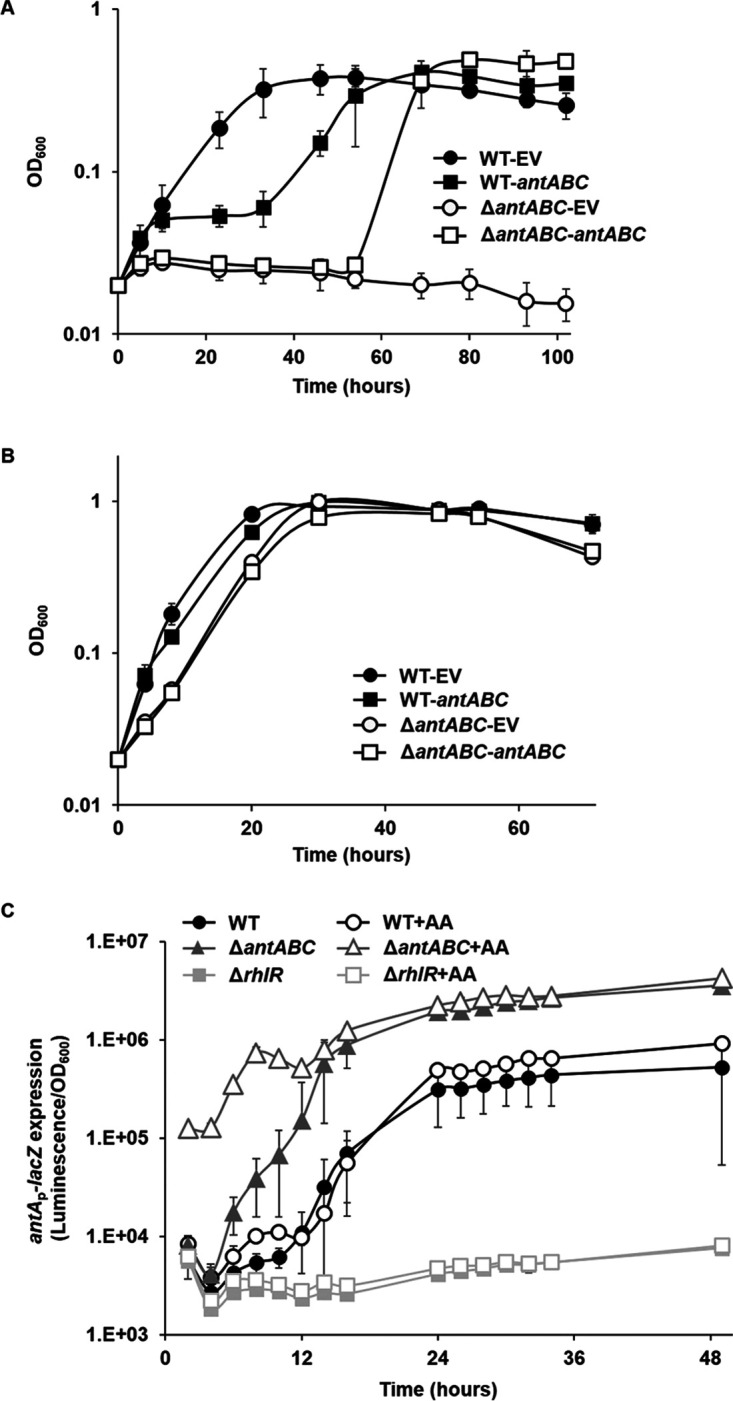
Effects of the early expression of *antABC* on growth. *P. aeruginosa PAO1* (WT) and *antABC* mutant (Δ*antABC*) that harbor pHJABC for the expression of *antABC* (*antABC*) or empty plasmid for the vector control (EV) were grown in MMA (A) or MMA supplemented with 0.2% glycerol (B). Cell growth was measured by OD_600_. (C) The WT, Δ*antABC*, and *rhlR* mutant (Δ*rhlR*) were grown in LB or LB supplemented with 0.1 mM anthranilate (+AA), and the *antA_p_-lacZ* expression was measured by β-galactosidase assay using the reporter plasmid (pJL201).

To better understand this, we introduced a plasmid-bearing arabinose-inducible *antABC* into P. aeruginosa and investigated growth with the overexpression of *antABC*. Interestingly, the addition of arabinose for inducing *antABC* sometimes shortened or abolished the intermediate lag in MMA when cells harbored only the empty plasmid, yet a long and clear intermediate lag was always observed when *antABC* was overexpressed (Fig. 6A). The overexpression of *antABC* in the *antABC* mutant caused a much longer intermediate lag in MMA, although growth was eventually restored (Fig. 6A). It was thought that arabinose may act as an alternative carbon source and prevent anthranilate from being initially degraded. To prove this, glycerol was supplied as an additional carbon source. The result showed that the intermediate lag was greatly alleviated in both the wild type and *antABC* mutant even when *antABC* was overexpressed (Fig. 6B). These results suggest that if anthranilate is present in abundance from the beginning of growth and degraded too early, it inhibits growth. For this reason, cells seem to have a mechanism to prevent anthranilate from being produced too early, and to keep the expression of *antABC* very low. When other nutrients are provided, cells seem to use those first and do not degrade anthranilate. In this situation, anthranilate does not appear to affect growth.

To prove this, the *antA*_p_-*lacZ* expression that reflects the AntR activity activated by intracellular anthranilate was investigated. As shown in Fig. 1B, the *antA*_p_*-lacZ* was greatly induced in the wild type when anthranilate began to accumulate, and much earlier in the *antABC* mutant. Interestingly however, even when anthranilate was exogenously added at the beginning of the culture, the expression of *antA*_p_*-lacZ* in the wild type did not accelerate or increase as much as in the *antABC* mutant (Fig. 6C), indicating that AntR cannot respond to the external anthranilate. Contrary to this, in the *antABC* mutant, the addition of anthranilate immediately induced the *antA*_p_-*lacZ* expression, indicating that if anthranilate cannot be degraded, AntR promptly responds to external anthranilate. This means that prior to the anthranilate peak, even if anthranilate is present in a high concentration outside the cell, AntR activity is suppressed in the wild type. This is most likely achieved through the degradation of anthranilate by *antABC*, yet the induction of *antABC* is limited to a small extent. This, on the other hand, means that this limited induction is sufficient to keep the AntR activity low. The rapid accumulation of anthranilate at the stationary phase, as was detected in the TLC analysis (Fig. S4), appears to occur with the release of this regulation, which leads to the high induction of *antABC*.

In the *rhlR* mutant, *antABC* expression did not increase at all even when anthranilate was added (Fig. 6C). This means that the release of this AntR suppression may be triggered by RhlR. Hence, in the *rhlR* mutant, the AntR-suppression continues and the intracellular anthranilate is maintained at a low concentration.

## DISCUSSION

The findings in this study can be summarized as follows. (i) P. aeruginosa temporarily accumulates anthranilate in the stationary phase (anthranilate peak). (ii) Anthranilate affects important physiological properties of P. aeruginosa, such as biofilm formation, virulence, and antibiotic susceptibility. (iii) Anthranilate affects not only P. aeruginosa itself, but also other bacteria around it. (iv) When anthranilate is degraded early in growth, cell growth is inhibited. All of these lead us to propose that anthranilate is a signal secreted by P. aeruginosa that can modulate the surrounding environment and neighboring cells. Above all, anthranilate satisfies the most important characteristic of signaling molecules in that it can change important physiological features and can be used to cross talk with other cells.

Anthranilate also has other basic features of signaling molecules. Like many other signals, it is produced at a specific growth phase and changes multiple phenotypes accordingly. In many cases, the production of signals and resulting phenotype changes occur in a bell shape at the point of entering the stationary phase: for example, competency in B. subtilis and Streptococcus (24, 25), and luminescence of Vibrio fischeri (26). The anthranilate peak is similar to the production pattern of these signal molecules, except that it occurs somewhat late after entering the stationary phase. Like most signal molecules, anthranilate is also a small diffusible molecule and has a receptor called AntR. While AntR has been found to regulate the expression of *antABC* by recognizing anthranilate and binding to the *antABC* promoter (2, 4), little is known about the physiological role of *antR*. Nevertheless, it is clear that anthranilate causes important changes in the physiology of P. aeruginosa and other bacteria. Therefore, anthranilate meets the basic requirements to be considered a signal.

Since anthranilate is a precursor for PQS synthesis, and it has been reported that QS significantly affects anthranilate production and *antABC* expression (2, 5), it’s thought that these effects by anthranilate appear by affecting PQS signaling rather than by anthranilate itself. However, for several reasons, we believe that signaling by anthranilate is distinct from PQS signaling. (i) The effect of anthranilate is opposite to the effect of PQS. Anthranilate signaling occurs in the direction of lowering virulence, antibiotic tolerance, and biofilm formation. However, PQS signaling occurs in the direction of increasing all of them. (ii) The anthranilate peak occurs in the late-stationary phase while general QS signaling appears before the beginning of the stationary phase. (iii) In Luria-Bertani (LB) medium, PQS generation does not increase even if anthranilate is added at 1 mM (3), which is a much higher concentration than what we used in our study (0.1 mM). We have also confirmed that the activities of the QS regulators, including PqsR, did not change significantly by the addition of anthranilate (data not shown).

The phenotypes that are altered by anthranilate are biofilm formation, virulence, and susceptibility to antibiotics. It is not clear whether they are all affected by the same mechanism. The effect on biofilm formation can be understood in that anthranilate disintegrates biofilm by inducing biofilm dispersal, but the effects on virulence and susceptibility to antibiotics are difficult to understand. One clue is that, although anthranilate can be used as a nutrient, it inhibits cell growth when provided as the only carbon source early in growth (Fig. S7A). This lag became much longer if cells were unable to keep the expression of *antABC* to a minimum early in the growth phase (Fig. 6A). In fact, anthranilate itself is not toxic to cells even at concentrations as high as 10 mM (12) and does not cause growth retardation in P. aeruginosa. Nevertheless, another study has shown that anthranilate can cause a similar lag period (9). The study showed that mutations in *mexI*, which encodes an efflux pump, caused a long lag period similar to our results and suggested that the cause of this lag is likely the accumulation of anthranilate or its metabolite. This is because the lag was alleviated when the synthesis of anthranilate was blocked, and the lag became more severe when the conversion of anthranilate to PQS was blocked (9). Like our results, their results show that if low intracellular anthranilate levels are not maintained, toxicity occurs and inhibits cell growth. Additionally, the pump mutants they used showed peculiar phenotypic changes in growth, antibiotic susceptibility, and virulence that are the same phenotypes affected by anthranilate.

We believe that this anthranilate-mediated toxicity can be interwound with antibiotic susceptibility and virulence. Our previous study showed that antibiotic treatment as well as other conditions that induce growth inhibition increased the activities of QscR (a repressor of the QS response), SoxR (an oxidative stress sensor), and AntR in P. aeruginosa (27). This suggests that the intracellular anthranilate level and *antABC* expression are increased in growth-inhibiting conditions. While it has been previously reported that antibiotics induce oxidative stress to kill cells (28), the mechanism and reason for the increase of anthranilate in growth-inhibited cells were difficult to understand, and at the time we did not know its importance. However, the results of this study imply that the early increase of intracellular anthranilate may be toxic to cells, and this toxicity can act with antibiotics synergistically to enhance the effectiveness of antibiotics. QscR is a QS receptor that acts as an antagonist of the QS response, so when QscR is activated, the virulence of P. aeruginosa decreases (29, 30). The fact that the conditions that enhance the AntR activity also increased the activity of QscR (27), suggests that in this situation, QscR may be activated and attenuate virulence.

The anthranilate peak eventually occurs in the stationary phase. This means that after a certain point, the cells no longer need to regulate the anthranilate level. Perhaps cells eventually adapt to the high-level anthranilate and its vigorous degradation by the highly induced *antABC*. We believe that this adaptation enables cells to show some sort of social behavior. In fact, once this adaptation occurs, anthranilate seems to be secreted out as waste. However, this means that anthranilate at this point is dedicated to signaling, which is an important feature of signaling molecules. Generating an anthranilate peak may induce some sort of physiological change in surrounding cells and favor the cells that are adapted to the high-level anthranilate. Consider a biofilm situation where P. aeruginosa will begin to form a biofilm after the anthranilate peak. If the surrounding cells are well adapted to high-level anthranilate, they will cooperatively degrade anthranilate to decrease its level and join in biofilm formation. However, if the cells are not adapted, their *antABC* expression will be limited, preventing them from degrading anthranilate well and resulting in their exclusion from the biofilm by the antibiofilm effect of anthranilate. In other words, cooperators who participate in the degradation of anthranilate are favored. In fact, it has been reported that cells in a biofilm show high *antABC* expression (14). Since most of the non-adapted cells are likely young cells, the anthranilate peak can act as a hurdle that P. aeruginosa must cross to join a biofilm in the microbial community. Similar situations can occur when a population is exposed to antibiotics, because the nonadapted cells are susceptible in the high-level anthranilate. This can cause a population with more antibiotic tolerant individuals to form. This is why anthranilate can be considered a signal in a sociomicrobiological perspective, and distinguishes it from environmental cues.

Finally, we would like to note three points: First, as we mentioned earlier, RhlR is likely to play an important role in the adaptation to anthranilate, because the AntR suppression persisted in *rhlR* mutants. Second, although anthranilate was added at the beginning of the culture, *antABC* was not immediately induced. This is also found in many QS-regulated genes, where they are only able to respond to signals during the stationary growth phase. These “late-response” genes have been hypothesized to be under the control of some unknown mechanism(s) preventing their early expression (31, 32). Third, since QS controls the production of many virulence factors (33–35), it has been thought that the low virulence of the QS mutant is due to the absence of these QS-regulated virulence factors. However, our results provide evidence that there is virulence that is not related to the direct regulation by RhlR (Fig. 3A and B). Similar claims have been made that even *lasR rhlR* double mutants still displayed residual virulence, although lacking in the production of virulence factors (35, 36). It remains to be unraveled why strong virulence appears at an early stage when anthranilate is added to the attenuated *rhlR* mutant.

## MATERIALS AND METHODS

### Bacterial strains, culture conditions, and plasmids.

The bacterial strains and plasmids used in this study are listed in Table S1. P. aeruginosa and other bacteria were usually grown in Luria-Bertani (LB; tryptone 10 g/L, yeast extract 5 g/L, and NaCl 5 g/L) medium at 37°C with vigorous shaking. For solid medium, agar was added at 1.5% (wt/vol). Bacterial growth was measured by optical density at 600 nm (OD_600_). In order to cultivate P. aeruginosa using anthranilate as the sole carbon source, a minimal medium containing anthranilate (MMA; M63 salt [KH_2_PO_4_ 3 g/L, K_2_HPO_4_ 7 g/L, (NH_4_)_2_SO_4_ 2 g/L], 1 mM MgSO_4_, and 10 mM anthranilate, pH 7.0) was used. In case of not being used as a carbon source, anthranilate was added to media at 0.1 mM. Antibiotics were used at the following concentrations: carbenicillin, 150 μg/mL; ampicillin, 100 μg/mL; tetracycline, 50 μg/mL; and gentamicin, 10 μg/mL for Escherichia coli and 50 μg/mL for P. aeruginosa. L-arabinose (0.4%) or IPTG (isopropyl-β-d-thiogalactoside, 1 mM) was used to induce protein expression.

### Δ*antABC* mutant construction.

The Δ*antABC* mutant was constructed by gene replacement using the *sacB*-based suicide plasmid pEX19Ap (Table S1), as previously described (37). Briefly, the up- and downstream regions of the *antABC* operon (about 500 bp) were amplified by PCR using specific primers (F-*antA*up, R-*antA*up, F-*antC*down, and R-*antC*down in Table S2) and inserted into pEX19Ap (XbaI-BamHI site for *antA* upstream and SacI-EcoRI site for *antC* downstream). The tetracycline resistance (Tc^R^) cassette amplified from pEX18Tc (Table S1) by PCR using specific primers (F-Tc and R-Tc in Table S2) was inserted into the BamHI-SacI site between the upstream and downstream regions. As a result, the entire region of the *antABC* ORF was replaced with the Tc^R^ cassette on pEX19Ap. This recombinant plasmid (pEX19*antABC*) was introduced into P. aeruginosa PAO1 by conjugation through E. coli SM10 (Table S1), and the chromosomal integration of the plasmid was selected on VBMM medium (trisodium citrate 3 g/L, citric acid 2 g/L, K_2_HPO_4_ 10 g/L, NaNH_4_PO_4_ 3.5 g/L, 1 mM MgSO_4_, 100 μM CaCl_2_, pH 7.0) containing 50 μg/mL tetracycline. The integrated part was then resolved on VBMM medium containing 5% sucrose, and the mutant was selected on a plate containing sucrose and tetracycline. Correct mutation was confirmed by PCR with specific primers (Table S2, Fig. S3) and no growth on MMA.

### Intracellular overexpression of *antABC*.

*antABC* was overexpressed using pHJABC that was constructed by inserting the DNA fragment including the whole *antABC* operon into pJN105 that has an arabinose-inducible promoter (Table S1). The insert was prepared by PCR amplification with specific primers (F-*antA* and R-*antC*, Table S2), digested with EcoRI-XbaI and ligated into the EcoRI-XbaI-digested pJN105 to make pHJABC (Table S1). L-arabinose (0.2%) was used to induce the *antABC* expression.

### β-Galactosidase activity assay.

β-Galactosidase activity was measured using the Galacto-Light Plus kit (Applied Biosystems, USA) according to the manufacturer’s protocol. The cell culture was measured for OD_600_ and mixed with 10% chloroform. After vigorous vortexing and incubation for 15 min, 10 μL aliquots of the supernatant were taken and mixed with substrate solution from the kit. After 1 h of incubation, 150 μL of Accelerator II of the kit was added and luminescence was promptly measured using a multiwell plate reader (Tristar LB941; Berthold). The luminescence was normalized by the OD_600_ value of the culture, and β-galactosidase activities are presented as luminescence/OD_600_.

### Measurement of anthranilate levels in media.

To measure the anthranilate levels in the P. aeruginosa culture media, the spent media (SM) were prepared as previously described (2). Each overnight culture of the Pseudomonas strains was inoculated initially at OD_600_ = 0.02 in LB and grown at 37°C with vigorous shaking. Samples were taken at each time point while cultivating. Cells were removed by centrifugation at 4°C, and the supernatant was taken and kept on ice for use. The anthranilate levels were measured by two different methods: E. coli dual plasmid reporter assay (2) and HPLC analysis (13). For the reporter assay, two compatible plasmids, pJL201 (*antA*_p_-*lacZ* fusion plasmid) and pJN105A (AntR-expressing plasmid), were transformed into E. coli DH5α and the transformants were grown up to OD_600_ = 0.5. Then, L-arabinose and SM were added at 0.4% and 10%, respectively, and incubated for 2 h, and β-galactosidase activity was measured. The concentration of anthranilate was calculated from the standard obtained from the β-galactosidase activity measured when pure anthranilic acid (Daejung, South Korea) of known concentrations was applied. For HPLC analysis, the SMs were extracted with an equal volume of ethyl acetate, and the ethyl acetate fraction was carefully taken and evaporated. The pellet was dissolved in methanol, injected into a C18 reversed-phase column of HPLC (Shimadzu, LC-20AT), separated in methanol gradient (flow rate = 0.5 mL/minute), and detected by a UV detector (wavelength, 210 nm). Anthranilate was quantified using the standard peaks from pure anthranilic acid.

### Measurement of intracellular anthranilate levels.

P. aeruginosa cells were cultivated in the same way as above, harvested by centrifugation at 4°C, and washed with phosphate-buffered saline (PBS) to remove the residual spent medium. The cells were resuspended in distilled water and lysed by using sonication. The lysed cells were extracted with an equal volume of acidified ethyl acetate and analyzed by thin-layer chromatography (TLC). The extracts were spotted on TLC plates (Silica gel 60 F_254_, Merck, Germany) and separated in dichloromethane/methanol 20:1 to a distance of 50 mm. After separation, the plates were air-dried and examined under UV light. 0.5 mM synthetic anthranilate was spotted on the TLC plate as a migration standard.

### Measurement of *antABC* expression in P. aeruginosa.

*antABC* expression was measured using the *antA*_p_-*lacZ* reporter as previously described (13). The pJL201 plasmid was introduced into P. aeruginosa strains, and the transformant cells were cultivated in LB medium containing carbenicillin. While growing, aliquots were taken and the β-galactosidase activities were measured.

### Biofilm analyses.

Biofilm analyses were performed as previously described (11, 13). Overnight cultures of cells were inoculated at OD_600_ = 0.06 into fresh M63 medium (M63 salt [KH_2_PO_4_ 3 g/L, K_2_HPO_4_ 7 g/L, (NH_4_)_2_SO_4_ 2 g/L], 1 mM MgSO_4_, 0.5% casamino acid, 0.2% glucose) on 96-well polystyrene plates and incubated at 37°C for 48 h without shaking. After the cell growth was measured for OD_600_, planktonic cells were poured out and the plate was washed with water and dried for 10 min. Then, 180 μL of crystal violet (0.1%, wt/vol) was added to each well and incubated for 10 min to stain the biofilm attached to the well surface. After a brief wash, the biofilm-staining crystal violet was dissolved in 200 μL of 30% acetic acid. Staining levels were assessed by measuring absorbance at 600 nm (A_600_). The data were normalized by cell growth (OD_600_). Flow-cell biofilm analysis was carried out as previously described (11, 13). P. aeruginosa cells harboring the green fluorescent protein (GFP)-expressing plasmid pAB1 (Table S1) were cultured overnight in LB, diluted to OD_600_ = 0.5, and injected into a flow-cell chamber (2 mm × 2 mm × 50 mm). After 1 h of incubation, 1% TSB (Bacto Tryptic Soy Broth, BD) containing 150 μg/mL carbenicillin and 1 mM IPTG flowed at 200 μL/minute through the flow-cell chambers. Biofilms in flow-cells were grown for 84 h under constant flow and observed by fluorescence microscopy, as described below.

### Biofilm imaging and quantification.

Biofilm images were obtained using confocal laser scanning microscopy (CLSM; Olympus, FV10i) or fluorescence microscopy (Zeiss, Axioskop FL). The excitation wavelength for GFP was 488 nm, and emission wavelength was 500 nm. The 3-dimensional images of biofilms were reconstructed from plane images by using Imaris 9.5.1 image analysis software. Fluorescence intensity of the flow-cell biofilm was quantified by COMSTAT 2.1.

### Bacterial susceptibility to antibiotics.

Overnight cultures of the Pseudomonas strains were inoculated initially at OD_600_ = 0.02 in LB and grown at 37°C with vigorous shaking. Anthranilate was added to LB at 0.1 mM. The aliquots were taken at 6 and 24 h for the samples before and after the anthranilate peak, respectively, and diluted to 3 × 10^4^ CFU/mL in phosphate-buffered saline (PBS; 0.137 M NaCl, 2.7 mM KCl, 10 mM Na_2_HPO_4_, 1.8 mM KH_2_PO_4_, pH 7.4). Other bacteria such as E. coli, S. enterica, Bacillus subtilis, and Staphylococcus aureus were cultured in LB in the same way. Since the growth rate of each species was different, samples were taken at an OD equal to the OD of the 6-h culture of P. aeruginosa (OD_600_ = 2.0) and diluted in the same way. The cell diluents were mixed with antibiotics for 2 h and 10 μL was spread on a LB agar plate for colony counting. Cell survival was presented as a series of percentages compared to the control without antibiotics (which corresponds to 100%). Antibiotics were used in the following concentrations: carbenicillin, 50 μg/mL (for P. aeruginosa), 5 μg/mL (for S. aureus), and 1 μg/mL (for E. coli, S. enterica, and B. subtilis); gentamicin, 1 μg/mL (for P. aeruginosa, E. coli, and S. aureus), and 5 μg/mL (for S. enterica and B. subtilis); tobramycin, 1 μg/mL (for P. aeruginosa, B. subtilis, and S. aureus), 0.5 μg/mL (for E. coli), and 5 μg/mL (for S. enterica); polymyxin B, 1 μg/mL (for P. aeruginosa), 0.5 μg/mL (for E. coli), 5 μg/mL (for S. enterica), and 50 μg/mL (for B. subtilis and S. aureus). MICs were measured as follows: the bacterial strains that were grown as above were diluted to OD_600_ = 0.0001 in LB on 96-well plates, mixed with antibiotics at the indicated range of concentration, and incubated at 37°C for 24 h. The MIC values were evaluated by visual inspection on cell growth after 24 h.

### Virulence assay.

The virulence factor production and real virulence of P. aeruginosa were measured using *Tenebrio molitor* larvae by injecting the culture supernatants or live bacterial cells, respectively, as described previously (19, 20). The aliquots were taken from the P. aeruginosa culture at 6 h and 24 h, and cells were removed by centrifugation at 4°C. The supernatants were filtered through a 0.2 μm filter (GVS Abluo Syringe Filter) and concentrated by a 10-kDa cutoff Centricon (Vivaspin, Sartorius). Five μL of the concentrated supernatants, insect saline (130 mM NaCl, 5 mM KCl, and 1 mM CaCl_2_) or LB were injected into *T. molitor* larvae using a syringe. Insect saline (IS) and LB were both used as controls. For the live cell injection, the cultured cells were diluted to 1.12 × 10^5^ CFU/mL in insect saline and 5.6 × 10^2^ CFU (5 μL) was injected into each *T. molitor* larva by syringe. The larvae were incubated in petri dishes at 25°C and the surviving larvae were counted every 24 h. Survival rate was presented as a percentage (%).

### Statistical analysis.

The Student's *t* test (two-sample assuming equal variances) was used to determine the significance of differences using Microsoft Office Excel. *P < *0.05 was considered significant. All experiments were carried out in triplicate and repeated at least twice independently.
