# Supplementary material for: Anthranilate Acts as a Signal to Modulate Biofilm Formation, Virulence, and Antibiotic Tolerance of Pseudomonas aeruginosa and Surrounding Bacteria
Source: Microbiol Spectr. 2022 Jan 12;10(1):e01463-21. doi: 10.1128/spectrum.01463-21 (PMC8754147; doi:10.1128/spectrum.01463-21)
Supplement: SUPPLEMENTAL FILE 1 — Supplemental material. Download SPECTRUM01463-21_Supp_1_seq3.pdf, PDF file, 1.0 MB [file spectrum01463-21_supp_1_seq3.pdf]

Anthranilate acts as a signal to modulate biofilm formation, virulence, and antibiotic tolerance of *Pseudomonas aeruginosa* and surrounding bacteria

Hyeon-Ji Hwang, Xi-Hui Li, Soo-Kyoung Kim, and Joon-Hee Lee*

Department of Pharmacy, College of Pharmacy, Pusan National University, Busan, 46241, South Korea

Table S1. Bacterial strains and plasmids used in this study.

Name	Genotype	References
Pseudomonas aeruginosa		
PAO1	A wild type strain of *P. aeruginosa*	(1)
Δ*antABC*	*antABC* mutant of PAO1, Tc^R	This study
PDO111	*rhlR*::Tn*501*, PAO1, Hg^R	(2)
Escherichia coli		
MG1655	A wild type strain of *E. coli* K-12	Lab. collection
DH5α	*supE44* Δ*lacU169* (*ϕ80 lacZ*Δ*M15*) *hsdR17 recA1 endA1 gyrA96 thi-1 relA1*	Lab. collection
SM10	*thi-1 thr leu tonA lacY supE recA*::RP4-2-Tc::Mu (Km^R)	Lab. collection
Salmonella enterica		
SL1344	A type of *S. enterica* serovar Typhimurium	Lab. collection
Bacillus subtilis		
ATCC6051	A wild type strain of *B. subtilis*	Lab. collection
Staphylococcus aureus		
RN4220	A wild type strain of *S. aureus*	(3)
**Small animals**		
T. molitor	Yellow mealworm	Lab. collection
**Plasmids**		
pEX19Ap	Ap^R; *oriT*$^+$ *sacB*$^+$, gene replacement vector with MCS from pUC18, not replicable in *P. aeruginosa*.	(4)
pEX18Tc	Tc^R; *oriT*$^+$ *sacB*$^+$, gene replacement vector with MCS from pUC18.	(4)
pEX19*antABC*	pEX19Ap with *antABC* gene interrupted by *tet* genes of pEX18Tc, Ap^R Tc^R	This study
pJN105	*araC*-pBAD cassette cloned in pBBR1MCS, Gm^R	(5)
pJN105A	*antR* orf in pJN105, Gm^R	(6)
pJL201	*antA*$_P$-*lacZ* fusion in pQF50, Ap^R	(6)
pAB1	*gfp-mut2* gene in pMF54, Ap^R	(7)
pHJABC	*antABC* orf in pJN105, Gm^R	This study

Tc^R, tetracycline-resistance; Hg^R, $HgCl_2$-resistance; Km^R, kanamycin-resistance; Gm^R, gentamicin-resistance; Ap^R, ampicillin and carbenicillin resistance.

Table S2. Primers used in this study

Name	Sequence (restriction enzyme site underlined)	
**Overexpression**		
F-*antA*	TGAAGAATTCACCCATAACGACAACGCACAAG	*Eco*RI
R-*antC*	CCTCTCTAGAGCGCATCAGTCGCCGC	*Xba*I
**Mutagenesis**		
F-*antA*up	TGGGTCTAGAGTTCGCTGCGCGCCA	*Xba*I
R-*antA*up	CGGGGGATCCGCTTAGTCGACGATGCGC	*Bam*HI
F-a*ntC*down	CTCAGAGCTCGTCGCCGCCAGCTACCGGCAGGG	*Sac*I
R-*antC*down	GGAGGAATTCGGCCGGCTGGACATCCTGGT	*Eco*RI
F-Tc	TAATGGATCCTTAGACGTCAGGTGG	*Bam*HI
R-Tc	TGATGAGCTCCAATTCTTGGAGTGGTGA	*Sac*I
**Mutant confirmation**		
MC-F-*antA*up	ATTTTCGGGATGCGCATCGTCGAC	
MC-R-*antC*down	ATCCTTTTCCTCGCCTCCGACGAG	
MC-R-Tc	TGCTTCTCGCCGAAACGTTTGGTG	
MC-R-*antA*	AGAGATTGAGGTTGCGCAGGCG	

Fig. S1.

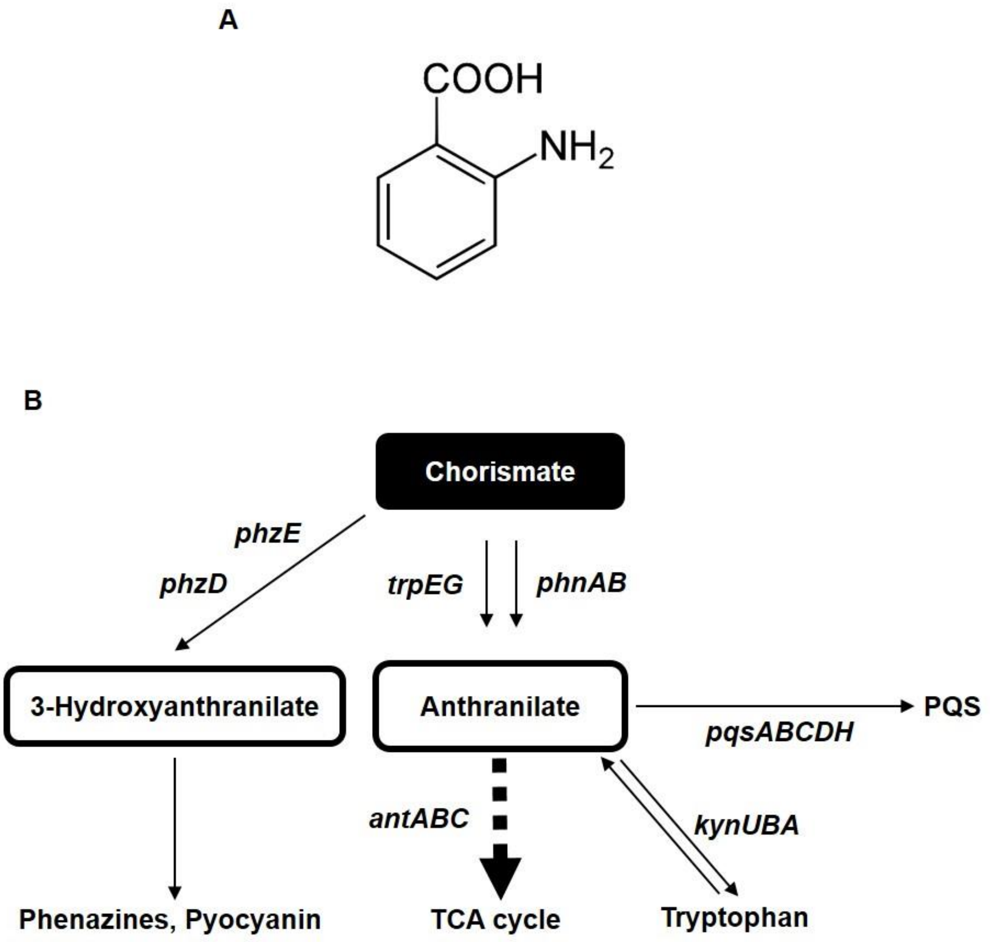

Fig. S1. Anthranilate, anthranilate metabolism, and related genes. The molecular structure of anthranilate (A) and the metabolic pathway around anthranilate (B) are shown. Anthranilate is a precursor for the synthesis of tryptophan and *Pseudomonas* quinolone signal (PQS), and metabolized by the anthranilate dioxygenase complex (*antABC* gene products) via the TCA cycle.

Fig. S2.

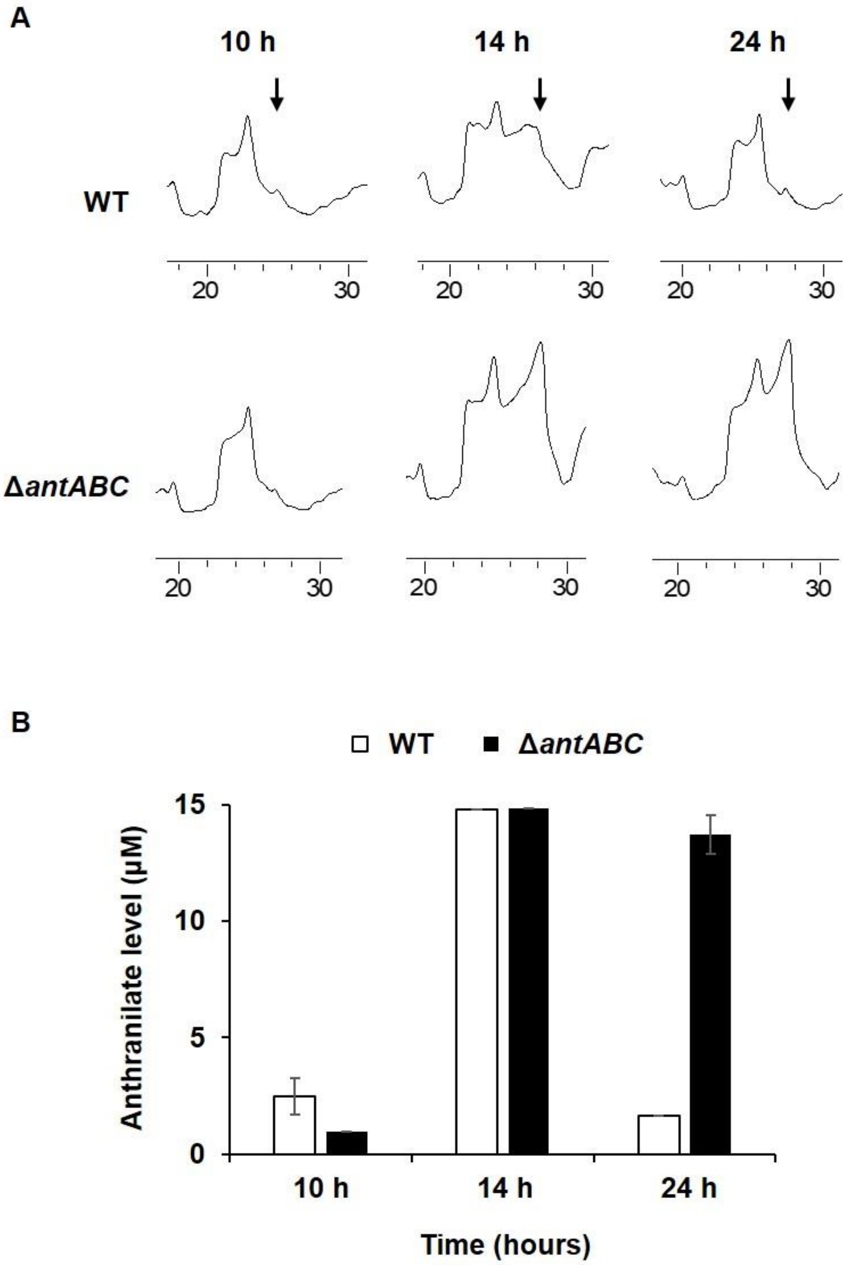

Fig. S2. Measurement of anthranilate levels by HPLC. *P. aeruginosa* cells were cultivated in LB broth, culture supernatants were taken at the indicated time points, and analyzed by HPLC analysis. The peaks for anthranilate were indicated by arrows (A) and quantified to be presented in graph form (B).

Fig. S3.

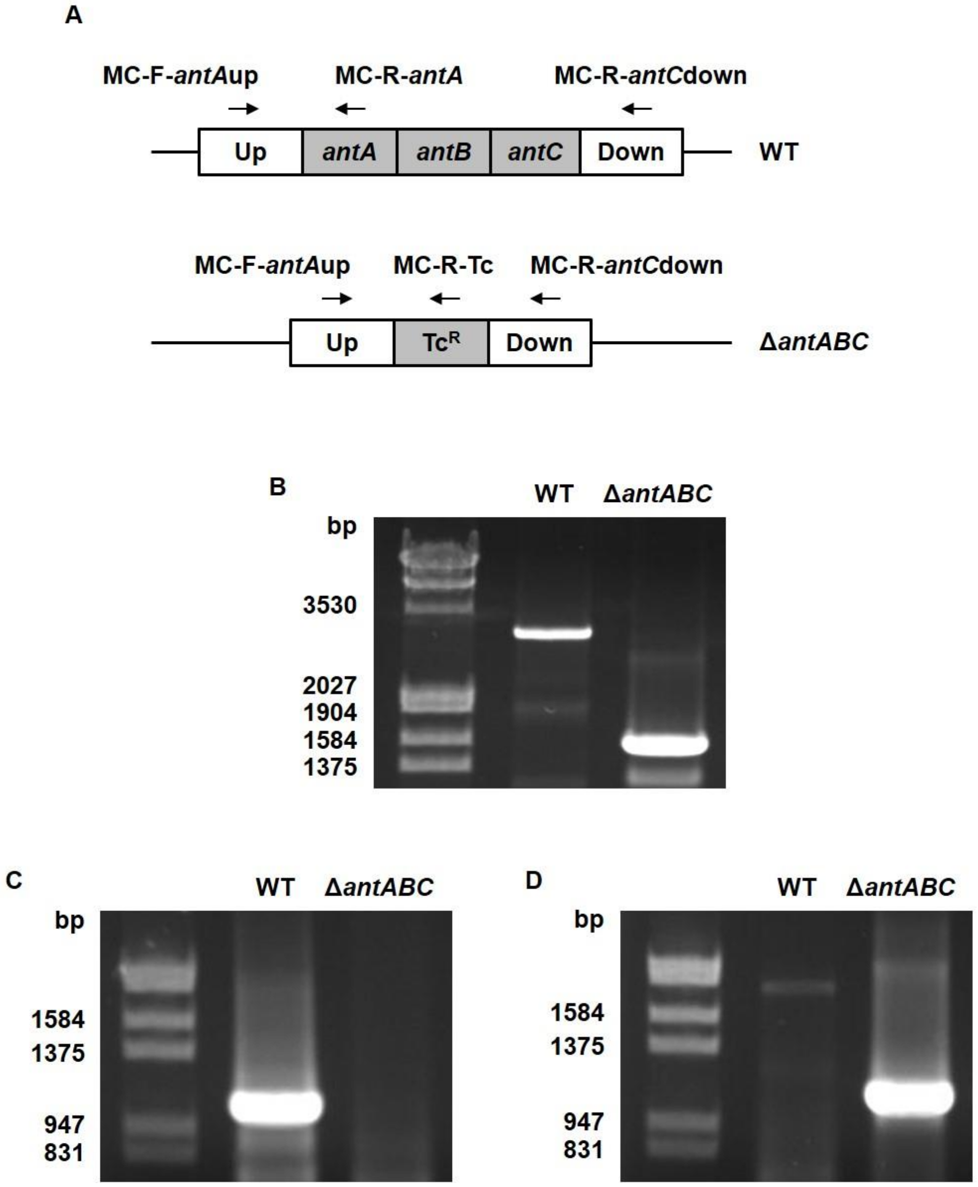

Fig. S3. Confirmation of the *antABC* deletion mutant. The location of the primers used in PCR analysis (A), and PCR results with the primer set of MC-F-*antA*up and MC-R-*antC*down (B), MC-F-*antA*up and MC-R-*antA* (C), or MC-F-*antA*up and MC-R-Tc (D). The expected sizes of amplified bands in each PCR reaction are follows; B, 3106 bp in the wild type (WT) and 1536 bp in *antABC* mutant (Δ*antABC*); C, 1054 bp only in WT; D, 1080 bp only in Δ*antABC*.

Fig. S4.

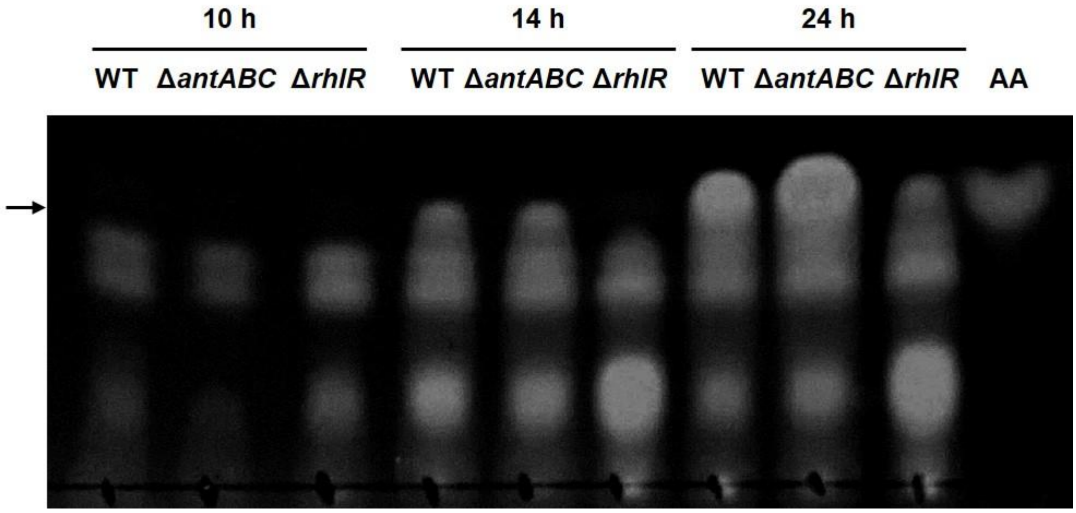

Fig. S4. Thin layer chromatography (TLC) analysis on the intracellular anthranilate levels. *P. aeruginosa* cells were grown in LB, harvested by centrifugation at 4℃, and washed with PBS to remove the residual spent medium. The cells were resuspended in distilled water, lysed by using sonication, extracted with an equal volume of acidified ethyl acetate, and analyzed by TLC. 0.5 mM synthetic anthranilate was spotted on the TLC plate as a migration standard. The spots for anthranilate are indicated by the arrow.

Fig. S5.

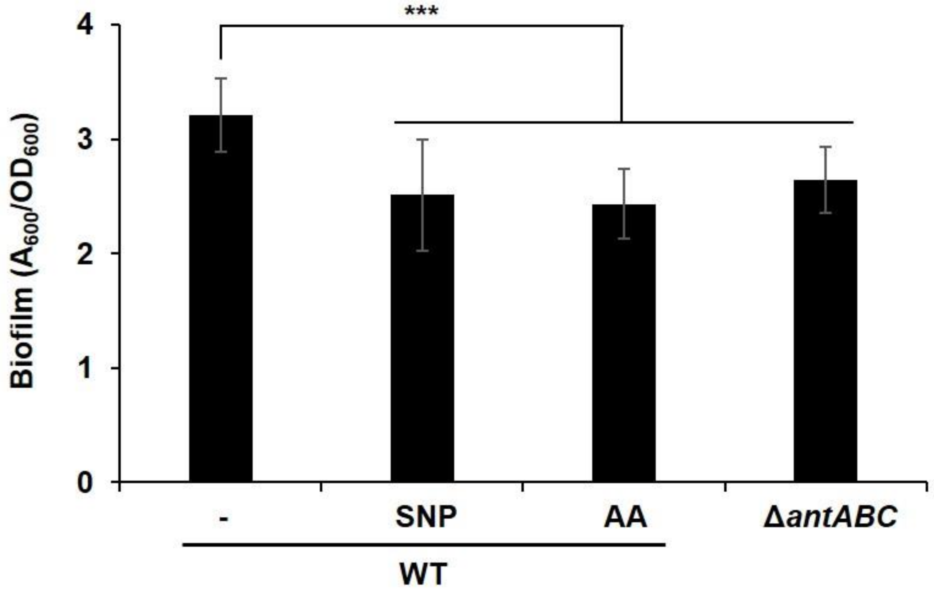

Fig. S5. The *antABC* mutant formed less biofilm than the wild type. Biofilm formation of the wild type (WT) and *antABC* mutant (Δ*antABC*) was measured by static biofilm assay as described in the Materials and Methods section. Sodium nitroprusside (SNP) and anthranilate (AA) were treated at 5 μM and 0.1 mM, respectively. ***, $p < 0.005$.

Fig. S6.

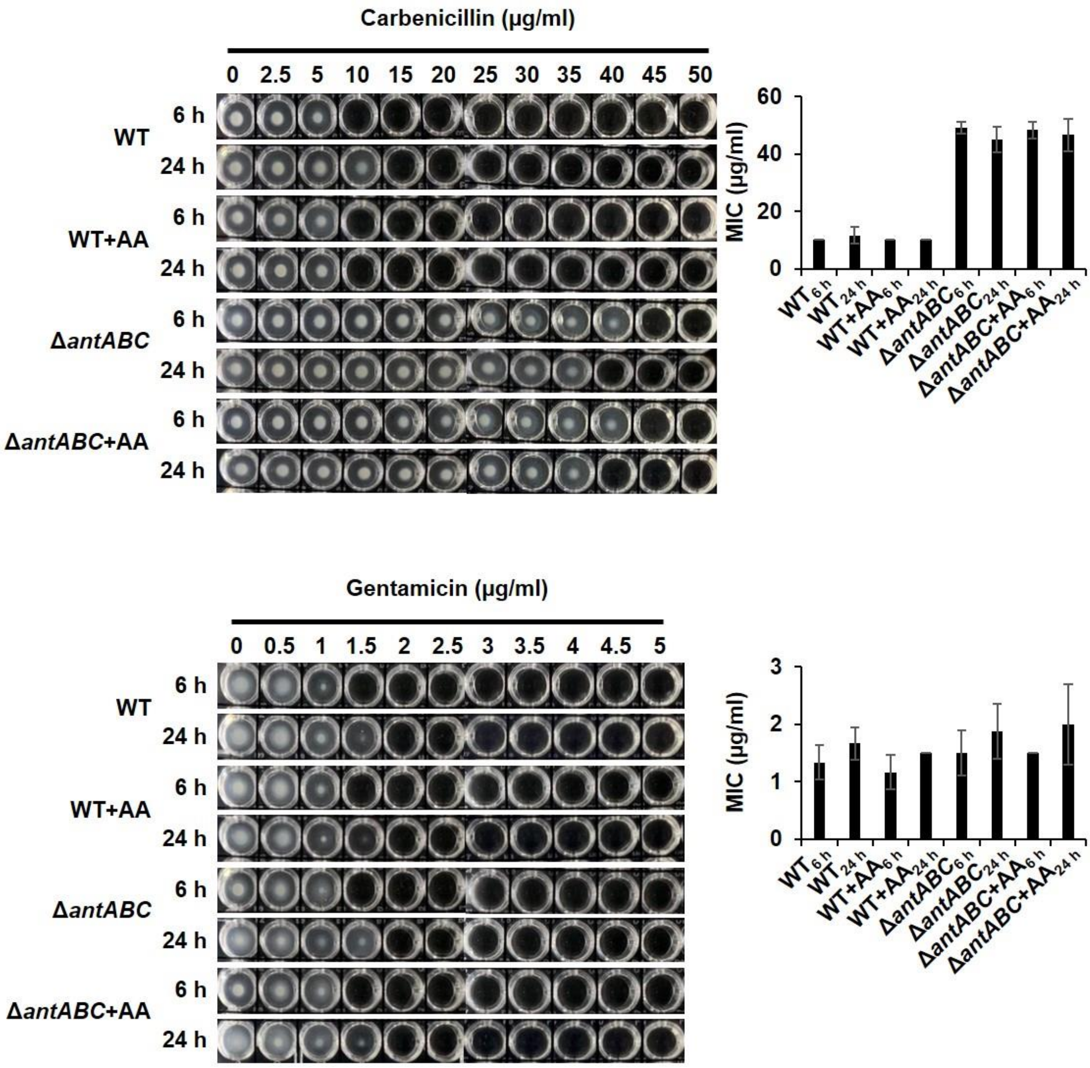

Carbenicillin (μg/ml)
0 2.5 5 10 15 20 25 30 35 40 45 50
WT
6 h
24 h
WT+AA
6 h
24 h
ΔantABC
6 h
24 h
ΔantABC+AA
6 h
24 h
MIC (μg/ml)
60
40
20
0
WT 6 h
WT 24 h
WT+AA 6 h
WT+AA 24 h
ΔantABC 6 h
ΔantABC 24 h
ΔantABC+AA 6 h
ΔantABC+AA 24 h
Gentamicin (μg/ml)
0 0.5 1 1.5 2 2.5 3 3.5 4 4.5 5
WT
6 h
24 h
WT+AA
6 h
24 h
ΔantABC
6 h
24 h
ΔantABC+AA
6 h
24 h
MIC (μg/ml)
3
2
1
0
WT 6 h
WT 24 h
WT+AA 6 h
WT+AA 24 h
ΔantABC 6 h
ΔantABC 24 h
ΔantABC+AA 6 h
ΔantABC+AA 24 h

Fig. S6 - continued.

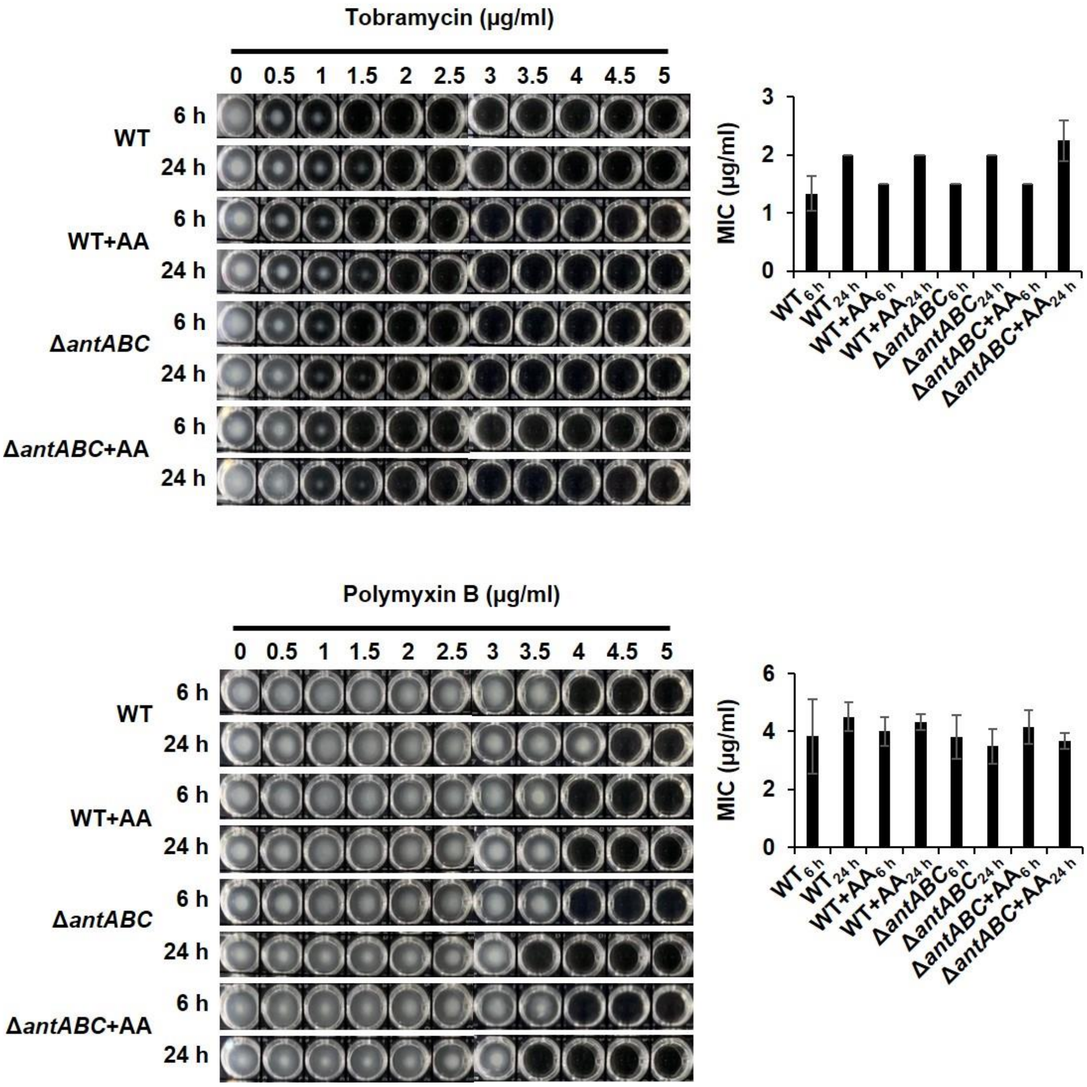

Fig. S6. Minimum inhibitory concentration (MIC) measurement with anthranilate. MIC was determined as described in the Materials and Methods and the results were presented as graphs. Anthranilate was added to the main culture media at 0.1 mM (+AA).

Fig. S7.

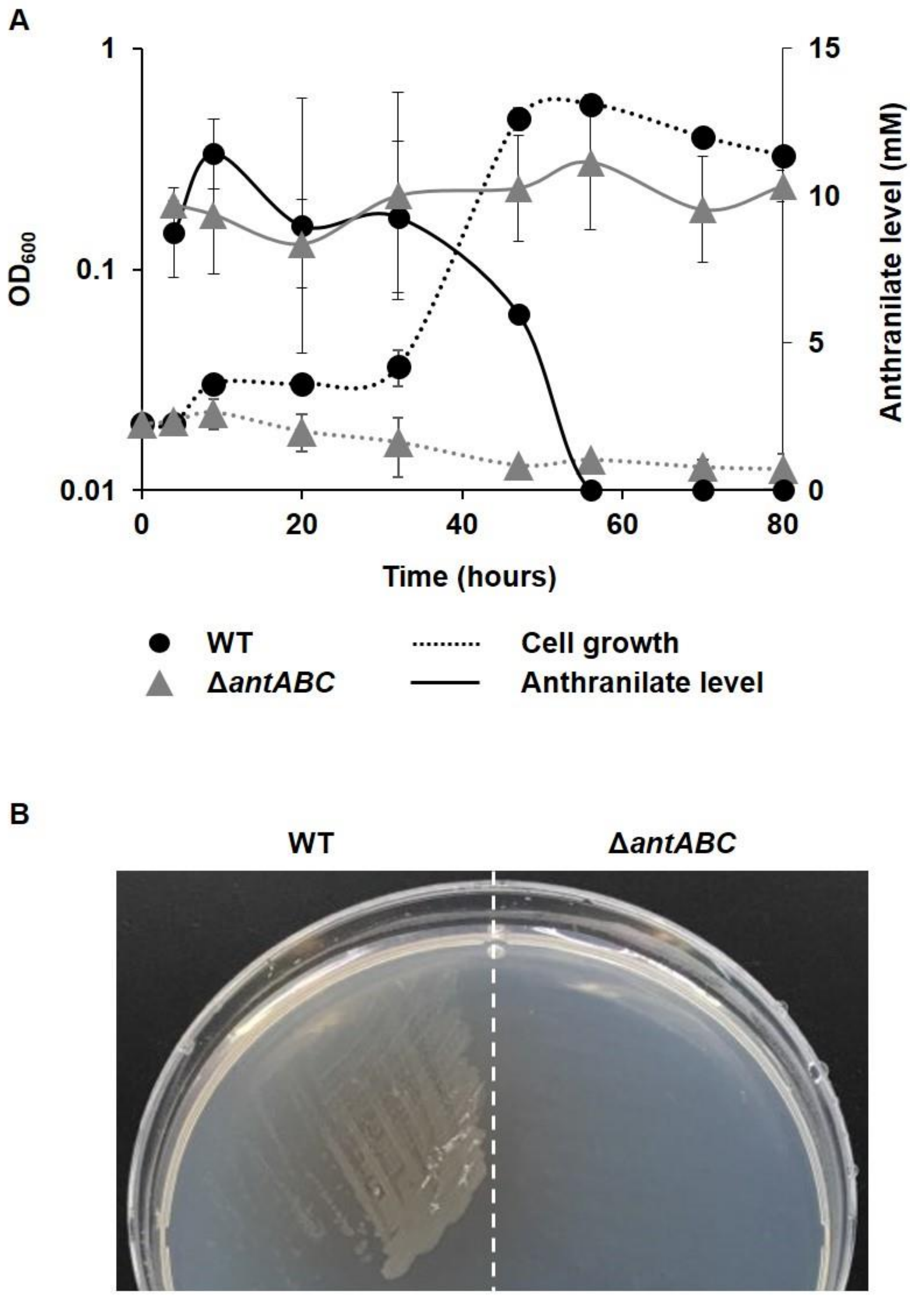

Fig. S7. Growth with anthranilate and degradation of anthranilate. *P. aeruginosa* wild type (WT) and the *antABC* mutant (Δ*antABC*) were grown at 37°C in MMA for 80 hours (A) or on solid MMA agar plate for 3 days (B). Growth was monitored by OD_{600} and the anthranilate levels in the spent media were measured by reporter assay.

Reference

1. Pearson JP, Pesci EC, Iglewski BH. 1997. Roles of Pseudomonas aeruginosa las and rhl quorum-sensing systems in control of elastase and rhamnolipid biosynthesis genes. J Bacteriol 179:5756-67.
2. Brint JM, Ohman DE. 1995. Synthesis of multiple exoproducts in *Pseudomonas aeruginosa* is under the control of RhlR-RhlI, another set of regulators in strain PAO1 with homology to the autoinducer-responsive LuxR-LuxI family. J Bacteriol 177:7155-7163.
3. Nair D, Memmi G, Hernandez D, Bard J, Beaume M, Gill S, Francois P, Cheung AL. 2011. Whole-genome sequencing of Staphylococcus aureus strain RN4220, a key laboratory strain used in virulence research, identifies mutations that affect not only virulence factors but also the fitness of the strain. J Bacteriol 193:2332-5.
4. Hoang TT, Karkhoff-Schweizer RR, Kutchma AJ, Schweizer HP. 1998. A broad-host-range Flp-FRT recombination system for site-specific excision of chromosomally-located DNA sequences: application for isolation of unmarked Pseudomonas aeruginosa mutants. Gene 212:77-86.
5. Newman JR, Fuqua C. 1999. Broad-host-range expression vectors that carry the L-arabinose-inducible *Escherichia coli araBAD* promoter and the araC regulator. Gene 227:197-203.
6. Choi Y, Park HY, Park SJ, Kim SK, Ha C, Im SJ, Lee JH. 2011. Growth phase-differential quorum sensing regulation of anthranilate metabolism in Pseudomonas aeruginosa. Mol Cells 32:57-65.
7. Walters MC, 3rd, Roe F, Bugnicourt A, Franklin MJ, Stewart PS. 2003. Contributions of antibiotic penetration, oxygen limitation, and low metabolic activity to tolerance of Pseudomonas aeruginosa biofilms to ciprofloxacin and tobramycin. Antimicrob Agents Chemother 47:317-23.
